# A perspective profile of ADCY1 in cAMP signaling with drug-resistance in lung cancer

**DOI:** 10.7150/jca.36614

**Published:** 2019-11-01

**Authors:** Ting Zou, Junyan Liu, Li She, Juan Chen, Tao Zhu, Jiye Yin, Xi Li, Xiangping Li, Honghao Zhou, Zhaoqian Liu

**Affiliations:** 1 National Institution of Drug Clinical Trial, Xiangya Hospital, Central South University, Changsha, Hunan, P.R.China; 2 Department of Clinical Pharmacology, Xiangya Hospital, Central South University, Changsha, Hunan, P.R.China; 3 National Clinical Research Center for Geriatric Disorders, Xiangya Hospital, Central South University, Changsha, Hunan, P.R.China; 4 Department of Orthopaedics, The First Affiliated Hospital of the University of South China, Hengyang, Hunan, P.R.China; 5 Department of Otolaryngology Head and Neck Surgery, Xiangya Hospital, Central South University; 6 Otolaryngology Major Disease Research Key Laboratory of Hunan Province; 7 Changsha, Hunan, P.R.China. Department of pharmacy, Xiangya hospital, Central South University, Changsha, Hunan, P.R.China

**Keywords:** ADCY1, cAMP, Lung cancer, Drug resistance, Signaling pathway

## Abstract

Adenylate cyclase 1 (ADCY1 or AC1) is a member of ADCY superfamily and was primarily found to be expressed in the brain. ADCY1 is responsible for catalyzing ATP to cyclic AMP (cAMP). As a secondary messenger, cAMP can regulate plenty of cellular activities. cAMP can perform its regulation in cellular transport through the binding to cAMP dependent protein kinases (PKAs), cAMP-activated guanine exchange factors (EPACs) and cyclic nucleotide-gated channels functioning in transduction of sensory signals (CNGs). Lung cancer is one of the leading factors of cancer-related death worldwide. Platinum-based chemotherapy is the first-line treatment for advanced lung cancer patients. In addition, surgical treatment, radiation treatment, and molecular targeted therapy are also therapeutic options for lung cancer patients in clinical settings. However, drug resistance and toxicity are the major obstacles that affect chemotherapy outcome and prognosis of lung cancer patients. And the therapeutic efficiency and adverse effects are varying with each individual. In recent years, investigations based on genetic sequencing have revealed the emerging role of ADCY1 mutations in affecting drug efficiency in various cancers such as lung cancer, esophageal cancer and colorectal cancer. The potential function of ADCY1 in chemotherapy resistance is of great importance to be noticed and investigated.

## Introduction

Lung cancer is one of the leading causes of cancer-related death worldwide in recent years 1. There are two main kinds of lung cancer, including small-cell lung cancer (SCLC) and non-small-cell lung cancer (NSCLC). NSCLC is the most common subtype of lung cancer which accounts for approximately 85% 2. However, most of the lung cancer patients were at advanced stages (stage Ⅲ or Ⅳ) when diagnosed, which were not suitable for surgery. It is a huge challenge for treatment, with a 5-year survival rate less than 18% 3, 4. Although there are multiple therapy strategies in treating lung cancer patients, such as surgery, chemotherapy, radiotherapy, molecular targeting treatment and immunotherapy 5-9, platinum-based chemotherapy is still the widely used first-line therapeutic regimen currently 10. However, the dose related toxicity and drug resistance are the main obstacles limiting the therapeutic efficiency of platinum-based chemotherapy, which are vary greatly among individuals 11, 12. More and more genetic polymorphisms have been identified to be associated with platinum-based chemotherapy toxicity and drug resistance 13, such as EGFR, eIF3a, ERCC1, WISP1, RAC1 14-17. All these shows that to find the potential biomarkers of drug resistance is of great importance.

Adenylate cyclase 1 (ADCY1 or AC1) is a member of ADCY superfamily and is primarily found to be expressed in the brain 18. It is located in 7p12.3 and contains 22 exons; the molecular weight of its protein product is 130kd. Current investigations of its function are mainly focused on brain, which found that ADCY1 is involved in various processes of the central nervous system 19. ADCY1 was found to be highly expressed in inner ear cells comparing with the outer ear cells, and was also observed ubiquitously in the stereocilia on both the outer and inner hair cell of mouse. It could also affect hearing function in human and zebrafish 20. ADCY1 was a main regulator of the cAMP signaling pathway 21. It was also discovered to participate in a dopamine D4 receptors (D4Rs) modulated signaling pathway activated by neuronal PAS-domain protein 2 (NPAS2), which can regulate the circadian sensitivity of retinal ganglion cells 22. Loss of ADCY1 function might lead to drastically-behavioral impairments in sensorimotor and social behaviors 23. ADCY1 was also reported to be associated with alcohol addiction, and depression from adolescence to adult as well as in postpartum motherhood, which were accompanied by a down-regulation of ADCY1 expression 24, 25.

There are several report about the relationship between ADCY1 and drug resistance. ADCY1 was investigated to be significantly associated with the overall survival of patients with melanoma, and plays an important role in the metastases of melanoma 26. What's more, ADCY1 was reported to be overexpressed in NSCLC tissues, which may related to the prognosis of NSCLC patients 27. ADCY1 was hypermethylated in glioblastoma and associated with the survival time of the patients with malignant glioma 28. It was also discovered to be high-expressed in multidrug-resistant cells, which means ADCY1 may related to the chemotherapy drug-resistance in esophageal carcinoma cells 29. This review will be of great value to attract the public's attention to ADCY1 and its status as a novel biomarker in treating lung cancer patients. However, the specific mechanism underlying ADCY1 in regulating drug resistance in lung cancer patients is complex and unknown. DNA double-strand breaks (DSBs) pathway plays an important role in drug resistance, and DNA damage response (DDR) pathway participates in the cis-platinum induced cytotoxicity in non-small-cell lung cancer cells 30. ADCY1 may regulate drug resistance mediated by its function in these processes widely recognized. For instance, ADCY1 was reported to be involved in DSBs in denuded oocytes, the mRNA expression of ADCY1 was up-regulated in these DSBs cumulus cells 31.

In this review, we summarized the recent advances of ADCY1 in lung cancer and its underlying mechanisms. First, we overviewed the relationship between ADCY1 and cAMP, as ADCY1 is a vital regulator which can catalyse ATP to cyclic adenosine 3', 5'-monophosphate (cAMP). cAMP is an important second messenger involved in diverse cellular responses including cell growth, cell differentiation, cell proliferation, apoptosis and metabolism. Then, we focused our eyes on the downstream effectors of cAMP signaling pathway adjusted by ADCY1, and their relationship with drug resistance in lung cancer. We also explored the three classical signaling pathways and other signalling pathways modified by cAMP in the regulation of drug resistance in lung cancer. Moreover, we summarized the probable lncRNAs modulated by cAMP which may contribute to drug resistance in lung cancer patients. Finally, we highlight the feasibility of ADCY1 as a potential biomarker to predict drug resistance, which may provide an important clue for future lung cancer treatments.

## The relationship between ADCY1 and cAMP

**Figure 1:**
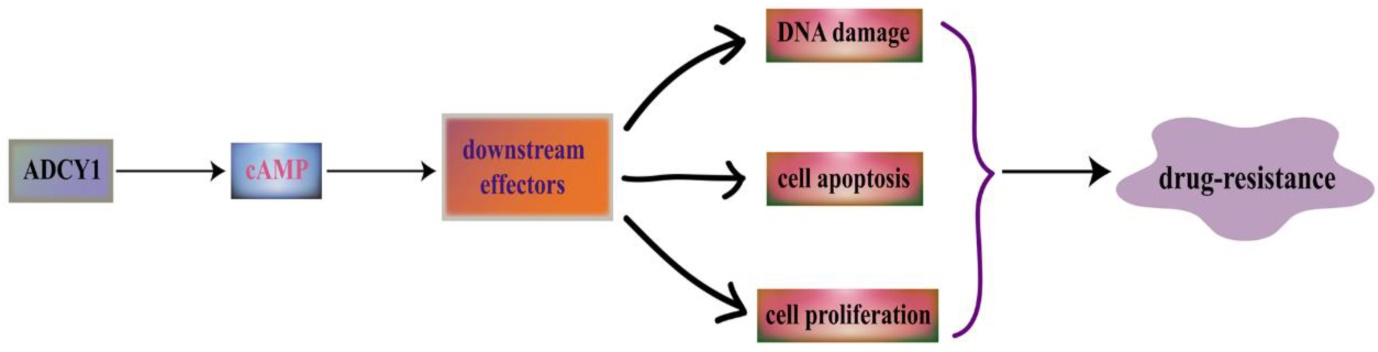
ADCY1 is a main regulator of the cAMP signaling pathway. ADCY1 can catalyse ATP to generate cyclic adenosine 3', 5'-monophosphate (cAMP), and then the 3', 5'-cAMP will degrade into 5'-cAMP by cyclic nucleotide phosphodiesterases. As a second messenger, cAMP participate in many important cellular activities. It can regulate its downstream effectors to have an influence on DNA damage, cell apoptosis and cell proliferation, which is linked with drug resistance.

ADCYs are activated by the stimulatory GTP-binding proteins (G proteins) which can response to various external signaling molecules through the regulation of G protein-coupled receptors, such as neurotransmitters and hormones 32. As a member of ADCYs, ADCY1 can catalyse ATP to cyclic adenosine 3',5'-monophosphate (cAMP), and then the 3',5'-cAMP will degrade into 5'-cAMP by cyclic nucleotide phosphodiesterases 33. cAMP can perform its regulation in cellular transport through the binding to cAMP dependent protein kinases (PKAs), cAMP-activated guanine exchange factors (EPACs) and cyclic nucleotide-gated channels (CNGs) 34. ADCY1 may affect DNA damage, cell apoptosis and cell proliferation through the regulation of the downstream effectors in cAMP signaling pathway (Figure 1).

PKAs and EPACs are the major targets of cAMP signaling pathway, activated PKAs-phosphorylated-enzymes can regulate relevant metabolism and transcription factors such as cAMP response element binding protein (CREB) 35. These cAMP sensors can modulate diverse physiological processes either alone or in cooperation with PKAs. EPACs can regulate cell proliferation, differentiation, apoptosis, and inflammation; they also play a critical role in lung-related diseases 36. Rap1 was identified as a main EPAC effector in several systems including lung 37. The EPAC/Rap1 signaling is associated with the complex network of EMT signaling; whose dysfunction may cause lung cancer 38. cAMP can activate CNGs to generate a depolarizing receptor potentially, it can also play an important role in the signaling of retinal photoreceptors and olfactory sensory neurons 39. CNGs are composed of two CNGA2 subunits and two modulatory subunits (CNGA4 and CNGB1b). They are participating in ligand-gated activation and regulation of rapid termination of odorant signal in an olfactory sensory neuron through the coordination with each other 40. Furthermore, PKAs, the main effector of cAMP, can participate in the regulation of cellular activities such as cell growth, proliferation, apoptosis and inflammation. EPACs can specifically activate the monomeric G protein Rap 41. CNGs are involved in the transduction of olfactory and visual signals. cAMP can perform its function in modulating diverse cellular activities through the regulation of these three main effectors (PKAs, EPACs and CNGs) 42.

**Figure 2:**
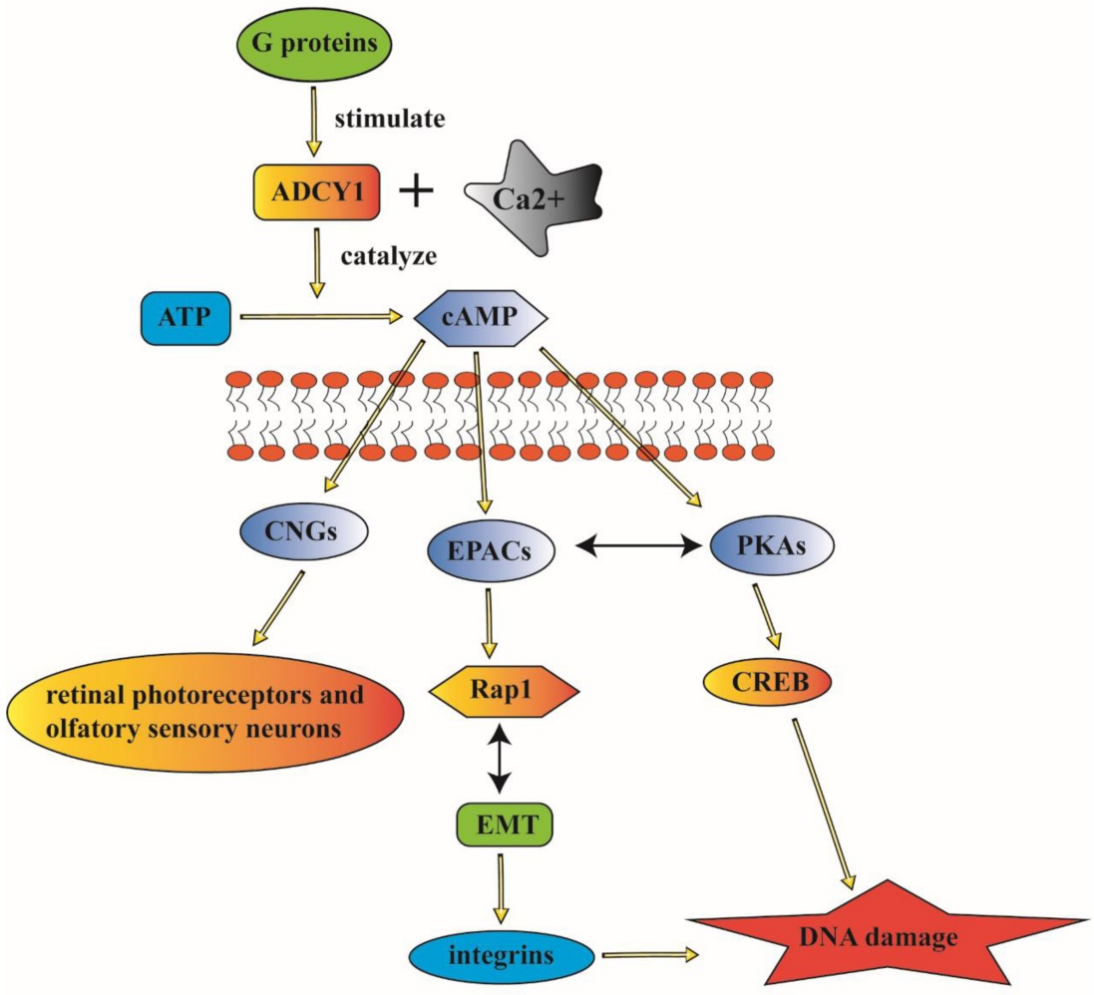
ADCY1 can participate in abundant cellular signaling pathways and contributes to physical behavior through the regulation of cAMP. ADCY1 can catalyze the formation of cAMP to modulate a series of signaling pathways. The major downstream effectors of cAMP including CNGs, EPACs and PKAs, which can regulate various cellular activities. And the mistaken control of ADCY1 as the upstream modulator can contribute to complex disorder of these regulation. The complicated signaling pathway indicate the probable association between ADCY1 and lung cancer.

ADCY1 can influence cAMP biosynthesis and degradation through the coupling by Ca^2+^. The interaction of ADCY1 with specific signaling proteins and regulatory factors can generate privileged domains in cAMP signaling pathways 43. And the overexpression of ADCY1 is associated with the enhanced activity of extracellular signal-related kinase (ERK)/mitogen-activated protein kinase (MAPK) signaling pathway 44. ADCY1 is a conversed element with a carboxyl tail, it can not only participate in the catalysis, but also is essential for membrane targeting due to its localization in the cytoplasm supporting cells. ADCY1 is engaged in abundant cellular signaling pathways and it can also contribute to physical behavior through the regulation of cAMP signaling pathway 45 (Figure 2).

## The association between cAMP and drug resistance in lung cancer

The cAMP signaling pathway plays an essential role in diverse cellular responses including cell growth, cell differentiation, cell proliferation, apoptosis and metabolism. cAMP signaling can also modulates cancer cell death induced by anticancer drugs and γ-rays 46. The normal regulation of DNA damage is of great significance, and its disordered process may lead to the cellular malignant transformation and tumor development. The DNA damage and repair mechanism is crucial for cell cycle progress 47, 48. Once the repair failed, the damaged cells are faced with cell death, including cell apoptosis. The abnormity of DNA damage and repair process can lead to cellular dysfunction and tumor development 49.

## The three classical signaling pathways modulated by cAMP to drug-resistance in lung cancer

cAMP has been identified to participate in response to various DNA damaging statuses. It has been widely acknowledged that cAMP can regulate cell apoptosis by modulating the expression of Bcl-2 family proteins, the inhibitor of apoptosis proteins (IAPs) and the repair protein (XRCC1) of γ-ray-induced DNA damage in human lung cancer cells 50.

cAMP signaling can decrease the phosphorylation of Bcl-2 through the inhibition of JNK activation, which will result in the down-regulation of cell autophagy and phosphorylation, and then reduce the ubiquitination and increase the expression of histone deacetylase 8 (HDAC8) 51. As an anti-apoptosis family member, Bcl-2 can inhibit apoptosis by suppressing essential proapoptotic proteins with multiple Bcl-2 homology domains 52. It can also modulate the permeabilization of the mitochondrial outer membrane (MOM), which is a critical step in apoptotic signal transduction, and then influence the cell survival of lung cancer cells. It has also been reported that the expression of Bcl-2 is associated with chemotherapy drug-resistance in malignant tumor patients. It has been reported that targeting Bcl-2 family proteins could overcome drug resistance in non-small cell lung cancer 53. It means that cAMP may also affect chemotherapy drug-resistance through the regulation of Bcl-2.

As a novel inhibitor of apoptosis proteins that inhibit apoptosis triggered by a variety of stimuli, IAPs can play important roles in suppressing cell death and regulating DNA damage in the cell cycle. The translation initiation regulation of the internal ribosome entry sites is one of the mechanisms that can regulate the expression of IAP 54. The cAMP-response-element-binding protein (CREB) can also regulate the activity of IAPs through the regulation of the enhancer sequence of IAPs to control cell apoptosis and DNA damage processes 55. The overexpression of IAP2 could enhance chemotherapy resistance and promote cell survival in lung cancer cells 56.

The X-ray repair cross-complementing protein 1 (XRCC1) can affect cellular sensitivity to ionizing radiation. The expression and polymorphisms of XRCC1 play an important role in DNA repair and it may be a prognosis biomarker for lung cancer patients treated with radiation or chemotherapy 57. The cAMP signaling pathway can regulate DNA repair and hence contribute to radiation and chemotherapy resistance by inhibiting the expression of XRCC1in lung cancer patients 58. The upstream signaling molecule of ADCY1 such as forskolin, an activator of ADCY1, can stimulate ADCY1 to catalyze cAMP, can inhibit DNA damage, DNA repair and cell apoptosis which is caused by the degradation of XRCC1 in an Epac-dependent pathway in lung cancer cells 59.

**Figure 3:**
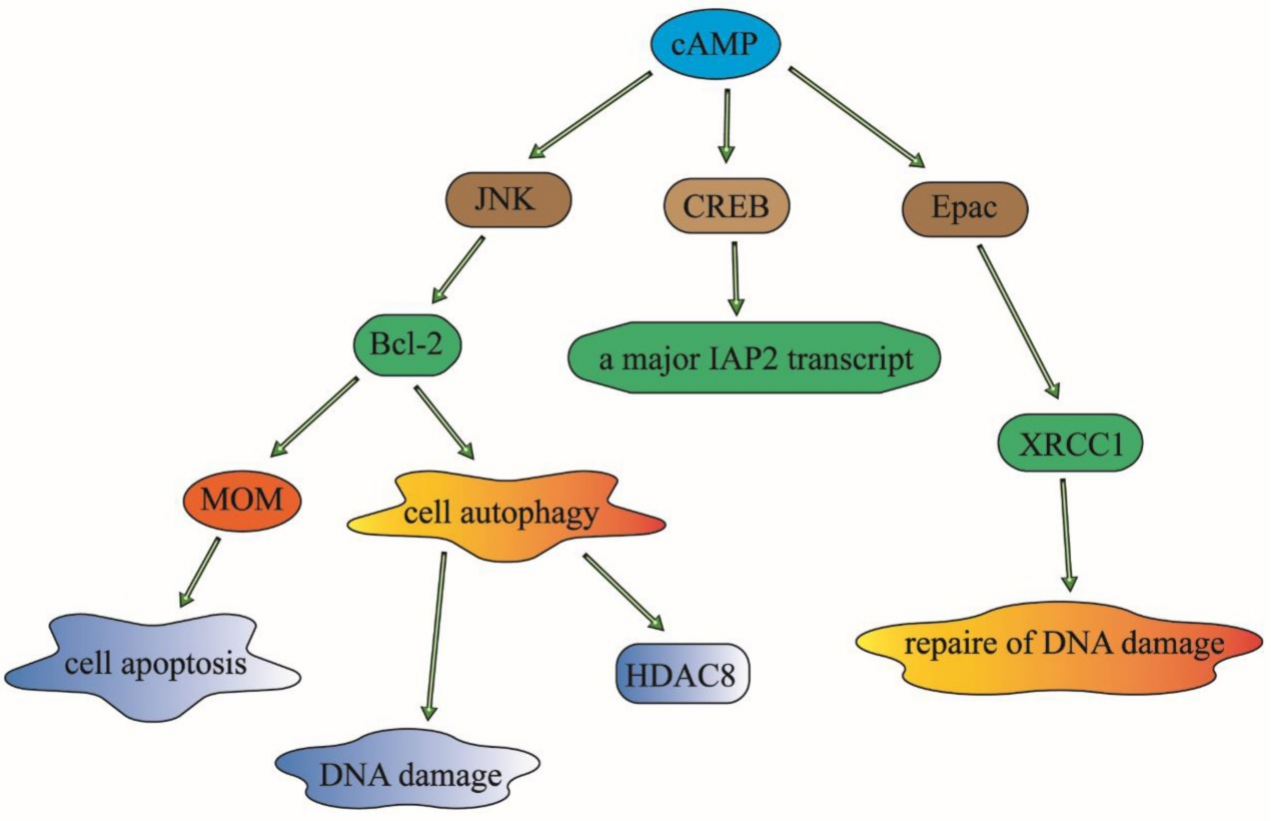
cAMP can modulate DNA damage, DNA repair and cell apoptosis to affect cellular survival or prognosis of patients with lung cancer. It can regulate the sensitivity to radiotherapy and chemotherapy in lung cancer patients, through the modulation of these three popular effectors: Bcl-2, IAPs and XRCC1. Given that these modulation of the three elements, cAMP can play an important role in the control of DNA damage and cell apoptosis. And then have an effect on the drug-resistance in lung cancer patients.

In conclusion, cAMP can modulate DNA damage and repair to regulate cellular survival and the prognosis of lung cancer patients. It may regulate the sensitivity to radiation and chemotherapy in lung cancer patients through the modulation of these three popular effectors: Bcl-2, IAPs and XRCC1 60 (Figure 3).

## Other signaling pathways contributing to drug resistance in lung cancer mediated by cAMP

Investigations have also revealed that cAMP can regulate radiation-induced DNA damage by other effectors. The ataxia-telangiectasia mutated (ATM) protein kinase plays a vital role in harmonizing the cellular response to radiation-induced DNA damage 61. cAMP can regulate DNA damage and enhance radiation-induced apoptosis in lung cancer cells through the inhibit of radiation-induced ATM activation by PKA-dependent activation of PP2A 62. Sirtuin 6 (SIRT6) was detected to be expressed abnormally in NSCLC tissues; it can regulate genomic stability and cell viability via removing acetyl groups from histones 63. cAMP signaling pathway can mediate the PKA-dependent inhibition of the Raf-MEK-ERK pathway to reduce the SIRT6 expression by promoting its ubiquitin-proteasome-dependent degradation, and then facilitate radiation-induced apoptosis in lung cancer cells 64.

Multidrug-resistance (MDR) phenotypes are a major obstacle to the efficiency of cancer. The ATP-binding cassette transporter MDR1/P-glycoprotein (MDR1/P-gp), as an ABC transporter, has been identified to be associated with MDR in various cancers, such as lung cancer, ovarian cancer, laryngeal cancer 65. There are some upstream modulators of cAMP participating in the regulation of MDR. As a 22 kDa Ca^2+^ binding protein, sorcin can induce the expression of MDR1/P-gp markedly through the activation of cAMP response element (CRE) located in the MDR1/p-gp promoter, and then regulate the CREB pathway which is associated with the MDR phenotype 66. The expression of MDR1/p-gp was also found to be upregulated in doxorubicin-resistant non-small cell lung carcinoma (NCI-H460) cells. CD56 is a cell adhesion protein which is a member of the immunoglobulin superfamily. CD56 has been reported to be associated with chemotherapy resistance (such as EGFR-TKIs) in lung cancer patients 67. cAMP can induce synaptophysin and chromogranin A, to increase the expression of CD56, which can make cells more sensitive to EGFR-TKIs (such as etoposide) and anthracycline compounds (such as DOX) by the regulation of cell adhesion 68.

MEK is activated in multiple cancer types such as non-small-cell lung cancer (NSCLC) and colorectal cancer (CRC). Selumetinib is a selective MEK1/2 inhibitor and is currently widely used in the treatment of NSCLC and SCLC 69. It has been reported that treatment with 8-CL-cAMP, a selective PKA inhibitor (PKAI), can reverse MEK resistance in NSCLC cells through regulation of MAPK. This is in line with the experimental evidence that PKA is an upstream regulator in the MAPK and EGFR pathway 70. There is also a functional link between EGFR/RAS-RAF-MEK-ERK pathway and PKA expression and activity. The cAMP and the PKA activity were also found to be high-regulated in the taxol-resistant cells, which have been recognized to be associated with taxol resistance in human ovarian carcinoma 71. OSU03013, a derivative of celecoxib, can compete with ATP to bind to cAMP-dependent protein kinase, and cause dephosphorylation of glycogen synthase kinase 3-beta (GSK3β), leading to beta-catenin (β-catenin) degradation 72. The interaction of GSK3β and β-catenin can regulate the invasion, proliferation and apoptosis of tumor cells, and modulate cisplatin-based chemotherapy sensitivity in lung cancer patients through the Wnt/β-catenin signaling pathway 73.

Parathyroid hormone-related protein (PTHrP)-(1-34) and PTHrP-(140-173) can protect lung cancer cells from apoptosis after the expose to radiation or chemotherapy drugs. A slight increase of cAMP was sufficient to protect lung cancer cells from apoptosis 74. PKA activation was identified to induce resistance to cell apoptosis and regulate cell survival through PTHrP. The expression of the cystic fibrosis transmembrane conductance regulator (CFTR) gene has been reported to be related to multidrug-resistance in lung cancer patients 75. It can increase in short-circuit current (Isc) in response to mediators through the cAMP-dependent Cl- secretion, which can mediate the activity of CFTR to modulate multidrug-resistance in lung cancer 76. There is an investigation found that the cyclic adenosine 3',5'-monophosphate (cAMP) analogue, 8-chloro-cAMP (8-Cl-cAMP), had a collateral growth-inhibitory effect on cis-diammine dichloroplatinum (CDDP) resistant cells, which means that 8-Cl-cAMP may be as a useful tool as cAMP to predict cis-diammine dichloroplatinum (CDDP) sensitivity 77. 8-Cl-cAMP has been shown to selectively bind to the site I receptor of the type II regulatory subunit (RII) of cAMP-dependent protein kinase, which means it is correlated with CDDP sensitivity in lung cancer 78.

**Figure 4:**
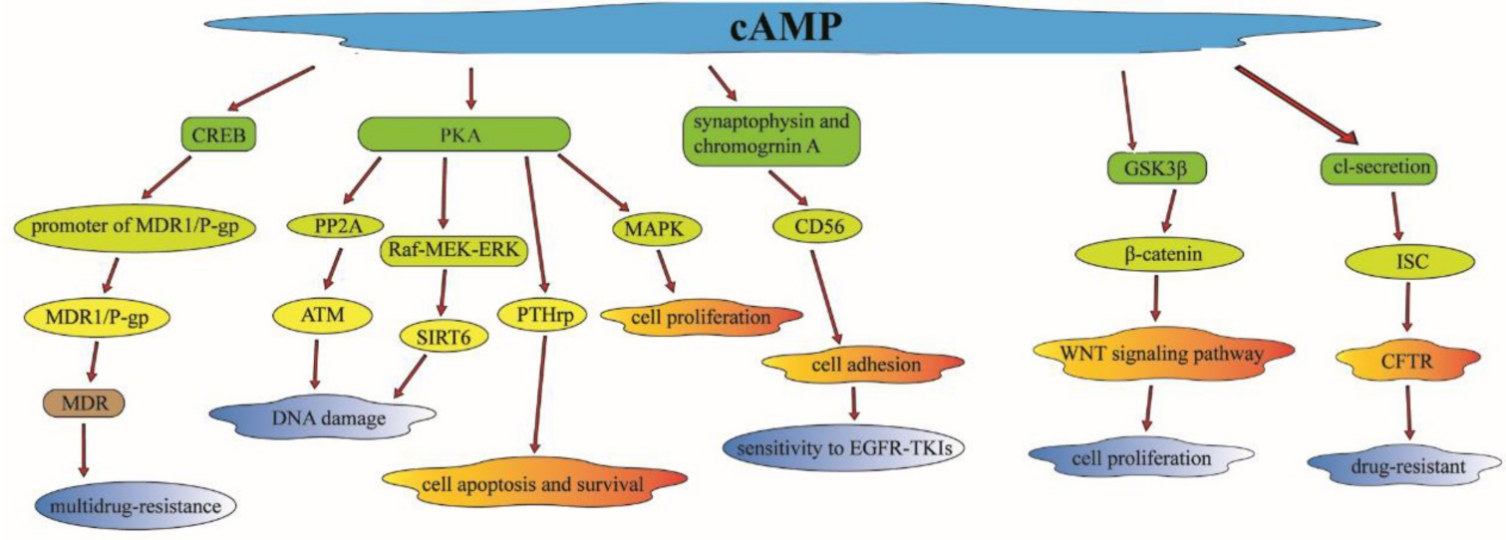
cAMP can regulate multidrug-resistance in lung cancer through the regulation of plenty of downstream effectors, which is participating in the cAMP signaling pathway such as the classical PKA and CREB or some other useful effectors. Through the regulation of these downstream effectors, cAMP can modulate cell apoptosis, cell survival, cell growth, cell cycle and cell proliferation which will have an effect on multidrug-resistance in lung cancer treatment.

In conclusion, cAMP or its analogue (8-Cl-cAMP) can regulate multidrug-resistance in lung cancer patients. There are plenty of downstream effectors participating in the cAMP signaling pathway such as the classical PKA and CREB. These complex but well-ordered signaling pathways can regulate the majority of cellular activities such as cell apoptosis, cell growth, cell cycle, cell proliferation, which may have an effect on multidrug-resistance in the treatment of lung cancer patients 79 (Figure 4).

## The association between cAMP and lncRNA to drug resistance in lung cancer

Long noncoding RNAs (lncRNAs) play a diverse range of biological roles, including regulation of gene expression. In recent years, lncRNAs have been recognized as emerging regulators in tumorigenesis 80. LncRNAs have been also identified to be involved in drug resistance, and hence found as potential biomarkers to predict prognosis of various cancers such as esophageal cancer, gastric cancer and lung cancer 81.

As a second messenger in multi-cellular activities, cAMP can regulate various lncRNAs effectors. COX-2 is an anticancer target; however, the drug resistance of COX-2 inhibitors is a major obstacle limiting their therapy efficiency in clinical treatment 82. cAMP can regulate the glycosylated COX-2 and PGE-2 to affect CRTC1 activation and LKB1 status, which means that cAMP can play an important role in the sensitivity to COX-2 inhibitors through the CRTC1 signaling pathway 83. LKB1 is a tumor suppressor gene which is frequently mutated and inactivated in NSCLC. Loss of LKB1 will promote cancer progression and influence therapeutic responses in preclinical investigations, due to its effect on drug efficacy through the regulation of MDR1 expression 84. cAMP can regulate LKB1 through cAMP-responsive element-binding protein (CREB) to affect the LncRNA LNC00473's activity, and then play an vital role in multi-drug resistance of lung cancer patients 85, given that overexpressed LINC00473 is correlated with poor prognosis, and stabilized expression of LINC00473 was required for cell proliferation and cell survival in LKB1-inactivated NSCLC cells. LNC00473 can also interact with cAMP/CREB through NONO, which is an element in cAMP signaling pathway 86. cAMP can also regulate LINC00473 to mediate decidualization of human endometrial stromal cells, which is through the cAMP-PKA pathway to modulate the phosphorylation of STAT3. It means that cAMP-PKA or cAMP-CREB can regulate the activity of LINC00473 through different pathways.

There are many LncRNAs highly up-regulated in liver cancer (HULC) known as Lnc HULC. They play important roles in tumorigenesis and metastasis. And they can be act as biomarkers to predict drug efficacy and prognosis of various cancers 87. cAMP can regulate Lnc HULC through a CREB binding site, and then modulate PRKACB activity through targeting the miR-372. cAMP can regulate tumorigenesis, cell survival and modulate multi-drug resistance in liver cancer through this signaling pathway 88. MEG3 is a long non-coding RNA which is a human maternally expressed gene associated with tumorigenesis and cell proliferation. Alteration of the expression of MEG3 is also correlated with cis-platinum resistance in lung adenocarcinoma. MEG3 can mediate chemosensitivity through the WNT/β-catenin signaling pathway by the regulation of p53, β‑catenin and survivin 89. cAMP can regulate the activity of MEG3 through a cAMP response element (CRE) in the proximal promoter region of MEG3, and then mediate cis-platinum resistance in lung cancer patients through regulating interactions between MEG3 and its downstream effectors 90.

**Figure 5:**
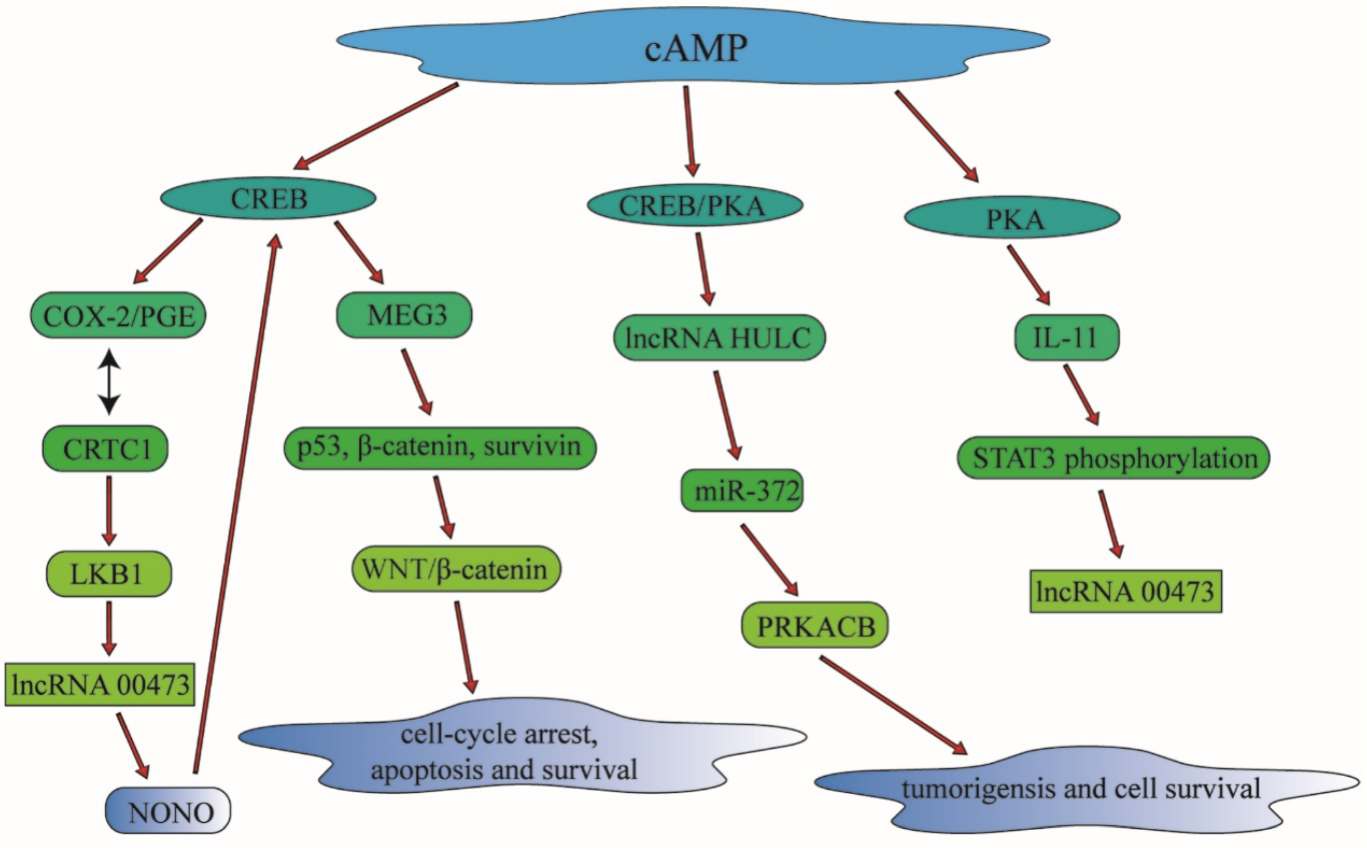
cAMP can control multidrug resistance in lung cancer and other malignant tumors through the regulation of particular lncRNAs involving in different signaling pathways, which is of great value in clinical treatment for lung cancer therapy. cAMP can modulate CREB and PKA to have an influence in the lncRNA such as lncRNA HULC and lncRNA 00473. Through the regulation of lncRNA, cAMP can also make a connection with cell apoptosis and cell survival, which is associated with drug-resistance significantly.

In summary, cAMP can regulate multidrug resistance in lung cancer and other malignant tumors through the regulation of particular lncRNAs involving in different signaling pathways, which is of great value in clinical treatment for lung cancer therapy (Figure 5).

## Conclusion

Here we reviewed for the first time of ADCY1 and its association with drug resistance in lung cancer. As a member of adenylate cyclase (ADCY), ADCY1 was first found to be expressed abundantly in brain and subsequent studies was mainly focused on its role in brain diseases. Mutations of ADCY1 were revealed to be associated with hearing impairment or other disorders in the nervous system. In this review, we focus on the function of ADCY1 in the regulation of multidrug resistance in lung cancer mediated by cAMP, which is a second messenger participating in plenty of cellular activities. cAMP has also been reported to be involved in the regulation of drug resistance in lung cancer through the downstream effectors in different signaling pathways such as the effectors belonging to DNA damage signaling pathway and specific lncRNAs. Taken together, ADCY1 is of great significance to be a novel biomarker to predict drug resistance in lung cancer patients. However, the specific mechanism of ADCY1 regulating drug resistance need to be further investigated.

## Abbreviations

EGFR: epidermal growth factor receptor
eIF3a: Eukaryotic translation initiation factor 3 subunit A
ERCC1: ERCC excision repair 1
WISP1: WNT1 inducible signaling pathway protein 1
RAC1: ras-related C3 botulinum toxin substrate
